# Pyropheophytin *a* in Soft Deodorized Olive Oils

**DOI:** 10.3390/foods9080978

**Published:** 2020-07-23

**Authors:** Raquel B. Gómez-Coca, Mahmoud Alassi, Wenceslao Moreda, María del Carmen Pérez-Camino

**Affiliations:** Department of Characterization and Quality of Lipids, Instituto de la Grasa-CSIC-, Campus of Universidad Pablo de Olavide, E-41013 Sevilla, Spain; mcperez@cica.es (M.A.); wmoreda@ig.csic.es (W.M.); mcperezcamino@ig.csic.es (M.d.C.P.-C.)

**Keywords:** chlorophyll pigments, olive oil, pheophytins, pyropheophytin, soft refining

## Abstract

Mild refined olive oil obtained by neutralization and/or by soft deodorization at a low temperature and its blending with extra virgin olive oil (EVOO) is not allowed and is difficult to detect. Chlorophyll derivatives, pheophytins and pyropheophytin, and their relative proportions were proposed as parameters to detect such processes. The objective of this study is to determine changes in EVOO, in terms of pheophytins and pyropheophytin, occurring after several well-controlled mild refining processes. The changes on those chlorophyll pigments due to the processes depend on the temperature, stripping gas, acidity and oil nature. The data obtained show that, at temperatures below 100 °C, the rate at which pyropheophytin *a* is formed (R_a_) is lower than the rate at which pheophytins *a+a’* disappear (R_a+a’_). As a consequence, the R_a+a’_ and R_a_ ratios are considered to be directly linked to pheophytins *a+a’* decrease instead of to pyropheophytin *a* formation. Stripping gas very slightly affects the transformation of the chlorophyll pigments; actually both acidity and N_2_ enhance the increment in the R_a+a’_ and R_a_ ratios. In relation to the oil nature, the higher the initial pheophytin *a+a’* content, the higher the increase in the R_a+a’_ and R_a_ relations.

## 1. Introduction

Olive tree (*Olea europaea*) is one of the most expanded crops in the world. This has repercussions not only regarding the nutritional point of view but also with respect to the economy of, mainly, Mediterranean countries. Both the International Olive Council (IOC) and the European Union consider virgin olive oil (VOO) as just the oil obtained from the fruit of the olive tree solely by mechanical or other physical processes under conditions, particularly thermal conditions, that do not lead to alterations in the oil, and which has not undergone any treatment other than washing, decantation, centrifugation, and filtration [1,2]. If the quality of the oil does not meet a number of standards [3], it cannot be considered as ‘edible’ and must be refined. Refined olive oil (ROO) is a flavorless, colorless product that cannot be sold by retail and that has to be mixed with genuine VOO. Controlled olive oil blends are available in the market under the designation of *olive oil* (OO) composed of refined and virgin olive oils. Blends of olive oil with other vegetable oils are available too [4]. However, the high market price that VOO can reach has made it target of both mislabeling and illegal blending, and much work has been done to uncover such practices, including that on the detection of the correct proportion of olive oil in legal blends with seed oils [5,6].

Regarding adulterations, they may consist, for instance, of the addition of hazelnut oil, of oil obtained from the second extraction of the olive paste (olive pomace oil), or of soft deodorized olive oil. Hazelnut oil can be detected within an interval of 20–25% through the determination of the difference between the actual and the theoretical content of triacylglycerols (TAG) with equivalent carbon number 42 (ECN 42) [7], and at lower percentages (5% and 2%, respectively) through the relations between different TAG [8] or through lupeol determination [9,10,11]. The presence of low-quality centrifugation oil (olive pomace oil) can be proven determining the wax content [12] and the aliphatic alcohols content [13] since they increase during the storage time previous to the second centrifugation [14]. As far as soft deodorized olive oils are concerned, they are still difficult, if not impossible, to detect, which has triggered considerable research on the subject.

Soft deodorized olive oils come from low quality oils with high acidity or weak organoleptic defects, which have been the objects of illegal practices channeled into concealing their negative flavor. One of the strategies commonly used consists of a neutralization (in case of high free acidity and/or very high bitterness) and/or soft deodorization at a low temperature (in case of low free acidity but negative sensory characteristics), and then blending with extra virgin olive oil (EVOO) [15]. This practice is difficult to detect since the conventional refining markers, such as stigmastadienes [16,17,18], trans fatty acids, ad TAG dimers [19], are only formed at high temperatures, such as those in standard deodorization in which the oil is kept at 180–250 °C for 30–180 min [15,20]. Actually, soft deodorization conditions are tailored to avoid the formation of those specific markers [21], as has been pointed out in recent studies conceived to resolve this problem. Actually, such studies use the determination of glyceridic compounds to detect the presence of soft deodorized oils in virgin olive oils. They focus on the fact that, after the application of mild refining conditions, the relationship between the diacylglycerol content and the free acidity breaks [20].

Furthermore, the determination of non-glyceridic components has also been used to define the effect of deodorization. Such is the case of the fatty acid alkyl ester (FAAE) content [15,22] that has been related with the oil’s sensory classification. In that case, it was demonstrated that such alkyl esters only proved the addition of soft deodorized oil when this had been extracted from fruits with fermentative defects (i.e., fusty, musty, and winey-vinegary), remaining unaffected in oxidized oils and in oils obtained from frozen olives [22,23]. Moreover, the effects of hydrolysis and oxidation have been utilized to detect the presence of soft deodorized olive oil in VOO [24] and so has been the determination of the composition of volatile compounds [21].

In any of the above-referenced methods, the results were not conclusive; therefore, there is a need for new markers. In this line and since soft deodorization passes at a certain temperature, it makes sense to focus on some other compounds sensitive to low-temperature treatments such as chlorophylls. Chlorophylls, the natural pigments responsible for the green color of vegetable oils [25], are highly sensitive and experience chemical and physical modifications after processing and storage [26]. The most widespread reaction is pheophytinization, which takes place by the breakdown and loss of the magnesium atom from the chlorophyll moiety to yield pheophytins (*phy*) [27]. One further step, as a result of the heat treatments, consists of the formation of pyropheophytins (*pyphy*) by the demethoxycarbonylation of the C13 atom [27].

It is clear then that there must be a connection between chlorophyll degradation products and the different stages of a soft deodorization process. Actually, Serani and Piacenti [28] used a correlation coefficient (cold index) to reveal the use of soft deodorization in virgin olive oils but, according to Gertz [29], the results were compromised by the method precision due to the fact that the cold index was influenced by the chlorophyll content of the oil and by the quantification of *phy* and *pyphy*, since the instability of chlorophyll hindered the possibility of using a calibration standard. Following those previous lines of research, we were convinced that the quantities and relations of *phy* and *pyphy* could be suitable markers for olive oil processing. Therefore, we decided to study how *phy* and *pyphy* were affected by soft deodorization and to establish the relationship between VOO chlorophyll pigment composition and the presence of soft deodorized oils, taking into account that the soft deodorization procedure may be tailored to suit the characteristics of the raw material [20].

## 2. Materials and Methods

### 2.1. Chemicals

All chemical reagents were of analytical grade. The standard of chlorophyll *a* was purchased at Sigma-Aldrich (Merck KGaA, Darmstadt, Germany). Sodium hydroxide pellets, phenolphthalein, and diethyl ether, were from Panreac Química, S.A.U. (Castellar del Valles, Barcelona, Spain). Acetone and methanol were from Romil Chemicals Ltd. (Waterbeach, Cambridge, GB, UK). The deionized water used was obtained from a Milli-Q 50 system (Millipore Corp., Burlington, MA, USA).

### 2.2. Samples

Four Spanish monovarietal olive oils (Hojiblanca, Picual and two Manzanilla samples) were purchased directly from producers. A second set consisting of six olive oils of several origins and qualities (L-1, M-1, H-1, L-2, M-2 and H-2), with no varietal specifications, was purchased from local markets or directly from producers.

### 2.3. Qualitative Analysis of Chlorophyll Pigments

Several methods have been proposed to determine chlorophyll pigments in olive oils, including rapid and routine techniques [28,30,31].

In this work, we follow the method described by the International Standard Organization [32] and the German Society for Fat Science [33], based on the procedure previously described by Gertz and coworkers [29]. This method is currently one of the most widely used for the determination of *phy* and *pyphy*. Briefly, 300 mg of the oil samples is weighed into a 4-mL vial and introduced, with the help of 1 mL n-hexane, into a 1-g silica solid phase extraction (SPE) column, previously activated with 5 mL hexane. Subsequently the vial is rinsed twice with 1 mL n-hexane and added onto the column. A first fraction is eluted with 5 mL of a mixture consisting of n-hexane:diethyl ether (90:10, *v*/*v*) and is discarded. A second fraction is eluted with 5 mL acetone and collected. Then, it is evaporated in a rotary evaporator and re-suspended in 0.5 mL acetone for its subsequent analysis using a high-performance liquid chromatography-diode array detector (HPLC-DAD).

The HPLC analyses of the chlorophyll pigments were carried out with an HP Agilent 1100 Liquid Chromatograph (Agilent Technologies, Santa Clara, CA, USA) equipped with a DAD. Acquisition of data was done with the Agilent ChemStation for the HPLC System program. The conditions for the HPLC assays were: Waters Spherisorb ODS2 C18 column (250 × 4.6 mm internal diameter, 3-μm particle size) (Waters Ltd., Hertfordshire, UK), 20-μL injection volume through a Rheodyne Manual Sample Injector Valve (Idex Health & Science LLC, Rohnert Park, CA, USA), and isocratic elution conditions water:methanol:acetone (4:36:60, *v*/*v*/*v*), at a flow rate of 1 mL/min. Sequential detection was performed at 410 nm.

### 2.4. Quantitative Analysis of Chlorophyll Pigments

Pigments were quantified with a calibration curve obtained by least-squares linear regression analysis. The concentration range of the curve fitted the expected level of chlorophyll in VOO. We proceed as follows:

From a 0.01% chlorophyll standard solution, we prepared five different diluted solutions in acetone (concentrations between 0.1 and 0.5 mg/kg) and we injected them, in duplicate, in the HPLC system.

The exact concentration of the aforementioned chlorophyll solutions (1.12 × 10^−7^ M, 2.24 × 10^−7^ M, 2.80 × 10^−7^ M, 4.48 × 10^−7^ M, 5.60 × 10^−7^ M) were determined spectrophotometrically at 410 nm, using the chlorophyll extinction coefficient (ε_410_ = 94700/M × cm) and molecular weight (Mw = 893.51 g/moL).

The relationship between the different chlorophyll pigments was calculated according to the following equations:

R_a+a’_ (%) = *pyphy a* × 100/(*pyphy a* + *phy a* + *phy a’*)

R_a_ (%) = *pyphy a* × 100/(*pyphy a* + *phy a*)

where *pyphy a*, *phy a* and *phy a’* stand for pyropheophytin *a*, pheophytin *a*, and pheophytin *a’*, respectively.

### 2.5. Sensitivity and Method Repeatability

Tests to assess the repeatability of the method and trials to establish the limit of detection (LOD) were performed according to published procedures [34].

The LOD can be defined as the minimum concentration of an analyte that can be detected, although not necessarily quantified, with an acceptable confidence through a given analytical procedure. These concentration values should produce sharp, symmetrical analyte peaks with no tailing or shoulders and with a signal-to-noise ratio of at least 3. That is a concentration whose signal equals the blank signal (Y) plus three times (*k* = 3) its standard deviation (*S*): LOD = Y + *k* × *S*.

In order to calculate the LOD, five olive oil solutions prepared at different dilutions from a sample with low chlorophyll pigments content were taken to the HPLC, their areas measured and the respective standard deviations calculated.

The repeatability of the method was assessed with three VOO samples of different chlorophyll pigment concentrations (L-1, M-1 and H-1 with low, medium and high chlorophyll concentration, respectively). We determined the *phy a+a’* and of *pyphy a* concentrations (in mg/kg) together with the R_a+a’_ percentage.

Measurements were done in triplicate. The statistical analysis of the repeatability was carried out following the ISO 5725 Norm [35] and AOAC Regulation [36].

The statistical parameters used were:

- S_r_: Standard deviation of the repeatability = $\sqrt{\frac{\sum {\left(x-\bar{x}\right)}^{2}}{\left(n-1\right)}}$
- r: Repeatability (2.8√S_r_^2^) or intra-laboratory variance.
- RSD_r_%: Relative standard deviation of the repeatability = 100 × Sr/mean.
- CI: Confidence interval (95%).

The statistical study of the results was carried out by one-way analysis of variance (one-way ANOVA) of a number of repeated samples. The minimum significant level was set at 5%. The analysis was performed using the SPSS 12.0 program (SPSS Inc., Chicago, WI, USA).

### 2.6. Olive Oil Soft Neutralization Process

We carried out the soft neutralization procedure using an aqueous sodium hydroxide solution at 12 % (*w*/*v*). In order to know the volume needed for the free fatty acid neutralization, we first determined the free acidity of the starting oil according to the method published by the IOC. This method drives to the calculation of the free acidity expressed as the percentage of oleic acid and its performance had already been tested according to the corresponding collaborative tests [37].

Next, we placed 10 ± 0.001 g of each olive oil sample in test tubes and added a volume of the 12% (*w*/*v*) aqueous sodium hydroxide solution corresponding to the free acidity, plus a 5% excess (2 mL approximately). We shook the tubes for 20 min and then centrifuged them (10 min, 3000 rpm, 16 cm centrifugation diameter). On each case, we discarded the aqueous phase and washed the remaining oily phase with 5–6 portions distilled water for 5 min. We repeated this last step until we had made sure there were no free-soaps (the pink color of the phenolphthalein disappeared completely). Finally, we centrifuge them for 10 min in the described conditions.

### 2.7. Olive Oil Mild Deodorization Procedure

Soft deodorization is a technique utilized to eliminate unpleasant odors in olive oil, getting a matrix that keeps its chemical composition unaltered. We carried out the process under soft thermal conditions, vacuum, and a certain stripping agent (N_2_ or Air), in a way that such gas passed for a given period of time through a volume of relatively hot oil at a low pressure. In order to do this, we prepared our own laboratory equipment, mimicking industrial conditions as much as possible. Such equipment consisted of the following parts:

1. Temperature controlled shaker.
2. Kitasato flask to prevent the sucking back of the sample.
3. Beaker with glycerine as thermal liquid and stirring magnet.
4. 60 mL Sample container with bubbler.
5. Thermostat.
6. Rotameter.
7. Stripping gas intake system.
8. Vacuum gauge.
9. Vacuum pump with vacuum control.

We took the olive oil samples through different mild deodorization processes (vacuum at 22.5 mmHg; 600 mL/min stripping gas), and studied the influence of the following factors (Table 1):

1. Deodorization time (using all four varieties: Treatment #1).
2. Deodorization temperature (hojiblanca and manzanilla 1 and 2 varieties: Treatment #2).
3. Free acidity (using picual variety).
4. Stripping gas (using hojiblanca variety: Treatment #3).

Moreover, the effect of combining neutralization and deodorization was considered. In order to do that, three olive oils with a low, medium and high content of chlorophyll pigments (L-2, M-2, and H-2, respectively) were used. After neutralization with sodium hydroxide (Section 2.6) and filtering, oils were soft deodorized under N_2_, at 98 °C, for three hours.

## 3. Results and Discussion

### 3.1. Sensitivity and Method Repeatability

The lowest detectable concentration of *pyphy a* was 0.07 mg/kg.

The data obtained in two consecutive determinations of the same sample, using the same analytical method, did not differ in more than the value of ‘r’ (Table 2). From those data (RSD_r_ = 0.34–6.59%, RSD_r_ = 2.5–10%, and RSD_r_ = 1.82–4.62%, for *phy* (*a+a’*) and *pyphy a*, and R_a+a’_, respectively) one may consider the method to have a good repeatability.

### 3.2. Qualitative Analysis of Chlorophyll Pigments

**Figure 1:**
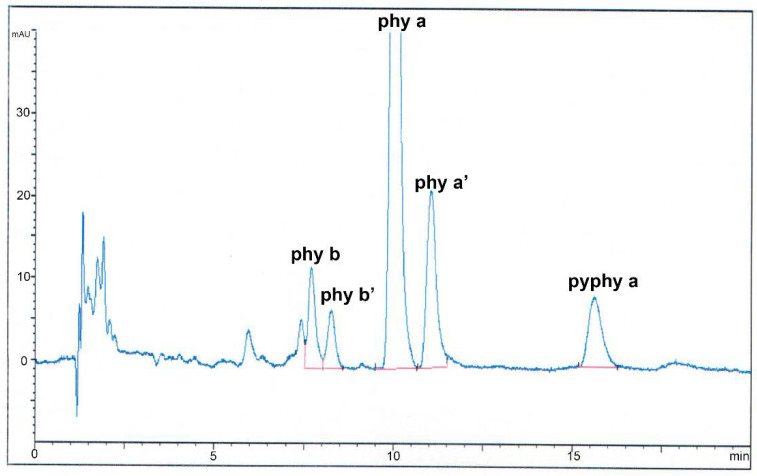
High-performance liquid chromatography (HPLC) chromatogram of the chlorophyll pigments of a soft deodorized olive oil cv. hojiblanca. From left to right, they correspond to pheophytin *b*, pheophytin *b’*, pheophytin *a*, pheophytin *a’*, and pyropheophytin *a*.

The selected conditions lead to the separation of individual pigments. The HPLC chromatograms consist of a series of peaks, three of them well resolved, whose retention times appear within the range from 15 to 25 min (Figure 1). They correspond to *phy b* and *b’*, *phy a*, *phy a’*, and *pyphy a*. After, *pyphy a’* might also appear. Those peaks were identified according to the published bibliography [28,33].

### 3.3. Quantitative Analysis of Chlorophyll Pigments

We calculated the analyte concentration corresponding to each peak (*phy a*, *phy a’*, and *pyphy a*) using the chlorophyll calibration curve: *concentration* (mg/kg) = −0.00319 + 0.0111 × *peak area*. We used chlorophyll as a standard instead of *pyphy a* because *pyphy a* is not commercialized as such and its synthesis is laborious.

### 3.4. Olive Oil Mild Deodorization Procedure

As shown in Table 3, the olive oil samples under study presented a wide variation in their chlorophyll content and, as observed in previous studies on different olive varieties [38], *phy a* was always the dominant pigment, being particularly high in the case of Manzanilla 1. According to our experience in the last ten years, where we have been analyzing 150 samples a year, on average, such a value may be considered to be very high. However, we have to keep in mind that it is not possible to give an expected average value (and therefore a reference value) for this parameter since the total content of chlorophyll compounds depends, among other things, on the characteristics of the starting samples and on the storage conditions [27]. Interestingly, the analyte concentrations seem to be dependent on the cultivar, which is the opposite to those observed by previous researchers over studies in which a higher number of cultivars were considered [39]. Therefore, the small number of samples advise us to be cautious regarding such a statement. We are aware that our assertion may seem contradictory to that observed in the cases of Manzanilla 1 and Manzanilla 2 (same cultivar but totally different results). In such a circumstance we have to take into consideration that the pigment composition and content of a certain oil is highly conditioned by the oil’s initial quality, light exposure, temperature, etc., and not only by the cultivar. This is indeed a line of research to be focused on during our next endeavors, where a wider number of varieties are being systematically analyzed.

#### 3.4.1. Effect of Deodorization Time

**Figure 2:**
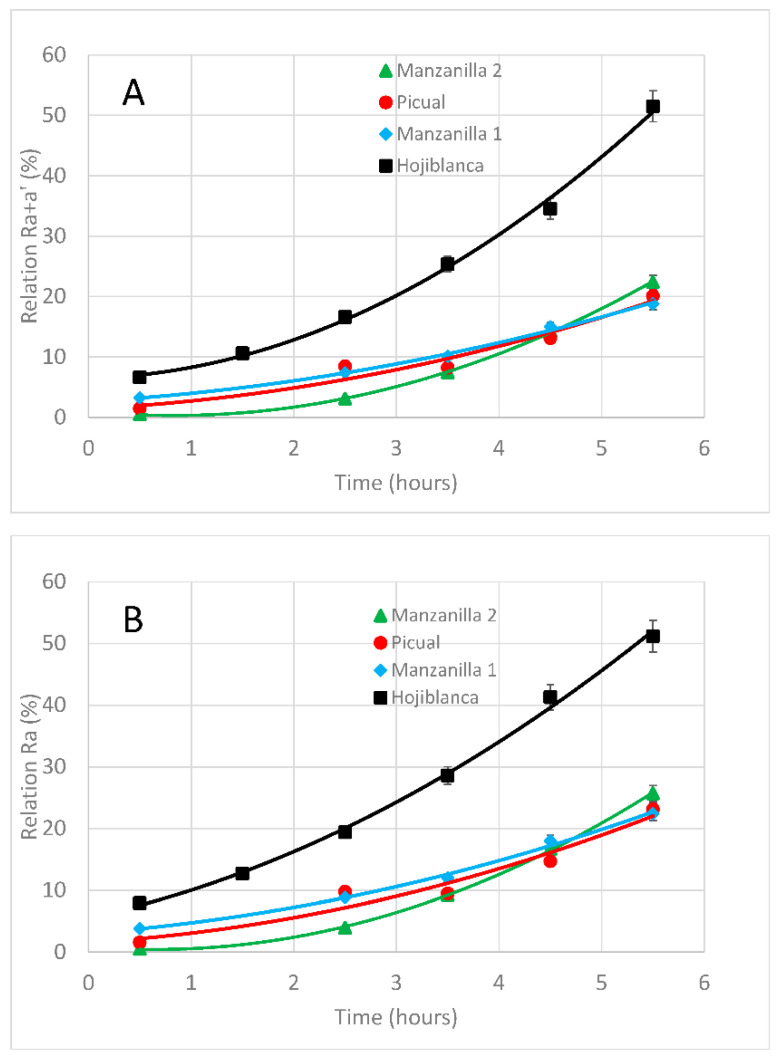
(**A**) R_a+a’_ relation vs. soft deodorization time and (**B**): R_a_ relation vs. soft deodorization time, in VOO samples from hojiblanca (squares), picual (dots) and manzanilla (triangles) cultivars. The soft deodorization process was carried out at 98 °C using N_2_ as a stripping gas. Each value corresponds to the average of two individual pieces of data.

In order to consider the effect of the deodorization time, samples of monovarietal VOO hojiblanca, manzanilla, (manzanilla 1 and manzanilla 2) and picual were subjected to different deodorization timespans, at 98 °C, using N_2_ as a carrier gas (Table 1, Treatment #1), for which results are shown in Figure 2. Under such accelerated conditions, the rate of evolution per hour is around 50% for hojiblanca and 25% for the others cultivars, which is very high in comparison with the normal 5–6% evolution per year observed during non-accelerated conditions [39].

In all cases, there was a quick rise in *phy a+a’* during the first hours of treatment, which slowed down later (Figure 2A). In the case of hojiblanca cultivar, the R_a+a’_ relation reaches 17% after around 2.5 h, whereas in the cases of picual, manzanilla 1 and manzanilla 2, at least 4.5 h are needed to exceed the 17% threshold. Such a 17% limit is the one proposed by Australian and Californian regulatory bodies and corresponds to the minimum *pyphy a* content accepted for fresh EVOO [40,41]. Differences among cultivars (Table 3) may be due to the low initial *pyphy a* content, 0.70 mg/kg, in comparison with the *phy a+a’* presence, 10.90 mg/kg, observed in hojiblanca, which in turn gives an already higher initial R_a+a’_ in comparison to the others.

The evolution observed in our study agrees with that previously published according to which the parameters of 100 °C and 60 min were considered as the optima, since they allowed negative volatiles removal and low *pyphy* formation (11.83%) [21].

If the *phy a’* content is not taken into account, that is, only R_a_ is calculated (Figure 2B), hojiblanca exceeds the 17% limit after around 1.5–2 h, whereas picual, manzanilla 1 and manzanilla 2 hold 4–4.5 h. It is then clear that the R_a_ relation may evidence the presence of soft deodorized oils in a better way than the R_a+a’_ relation does, meaning that *phy a* and *pyphy a* would reveal as key compounds to detect this kind of practice.

In any case, the increase in the R_a+a’_ and R_a_ relations is due to the *phy a+a’* reduction and not so much to *pyphy a* formation. This may be due to the *phy a+a’* destruction because of the effect of the deodorization conditions (98 °C), whereas *pyphy a* increases little after a certain time. We have to point out that we cannot expect an intensive *phy a+a’* destruction to be translated in an intensive *pyphy a* formation, since the concentrations of such derivatives do not keep a lineal relationship. Actually, previous studies show how, after the thermal treatment of olive oils, the disappearance of *phy a+a’* did not only correspond to the formation of *pyphy a* (and therefore to a lineal relationship) but also to that of other three products: 13^2^OH-phy a, 15^1^OH-lactone-phy a, and a colorless derivative [27], giving a more exact glimpse on the fate of *phy a+a’*.

#### 3.4.2. Effect of Deodorization Temperature

The effect of the deodorization temperature was studied with the monovarietal EVOO hojiblanca, manzanilla 1 and manzanilla 2. In this case, oils were subjected to two-hour length deodorizations at 50, 75, 100, 130, and 150 °C using N_2_ as a stripping gas (Table 1, Treatment #2).

Samples of hojiblanca and manzanilla 2 oils had relatively low initial concentrations of chlorophyll pigments (11.60 and 15.74 mg/kg, respectively), whereas, in the case of manzanilla 1, the pigment concentration was much higher (118.97 mg/kg).

**Figure 3:**
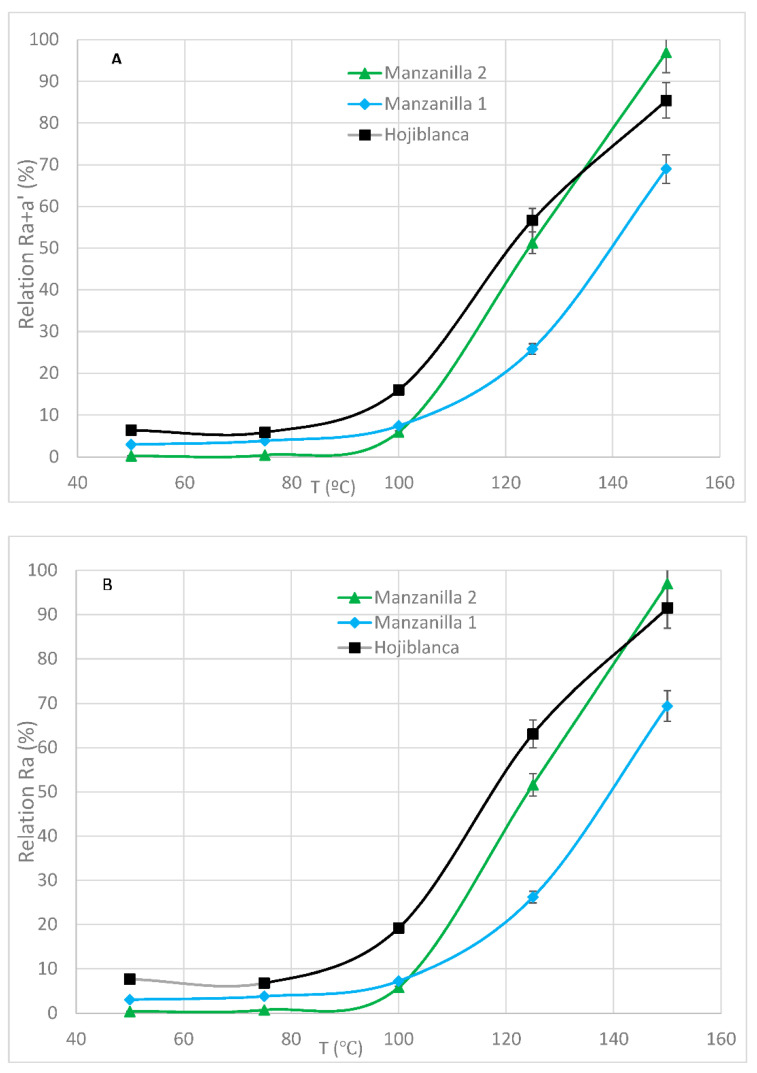
(**A**): R_a+a’_ relation vs. soft deodorization temperature and (**B**): R_a_ relation vs. soft deodorization temperature, in VOO samples from manzanilla and hojiblanca cultivars. The soft deodorization process lasted two hours. N_2_ was used as a stripping gas. Each value corresponds to the average of two individual pieces of data.

The results are shown in Figure 3. When one compares the R_a+a’_ and R_a_ relations between the three different samples, it is clear that the higher the initial pigment concentration, the higher the R_a+a’_ and R_a_ increments. Besides, increases in temperature lead to increases in both R_a+a’_ and R_a_ proportions, the latter being steeper than the former, meaning that not all *phy a+a’* turn into *pyphy a*. Besides, it is clear that there is not a linear correlation with temperature and that at temperatures below 100 °C the formation of *pyphy a* takes place slowly, as has already been observed before [21], although we demonstrate that a two-hour deodorization versus a one-hour timespan, as stated earlier [21], has no effect on *pyphy a* formation, temperature being the key factor. From 100 °C on, *pyphy a* formation goes up notably.

#### 3.4.3. Effect of Free Acidity

The study of the influence of the free acidity on the chlorophyll pigments during deodorization was carried out on samples of VOO from the picual variety. This parameter was chosen because of its relationship with the oil’s initial quality. Picual samples had 0.19% free acidity and were spiked with oleic acid in order to get aliquots with 2.0 and 5.0% free acidity. Samples were subjected to 2 to 5 h length deodorizations at 98 °C, using N_2_ as a stripping gas (Table 1, lines 11–13).

**Figure 4:**
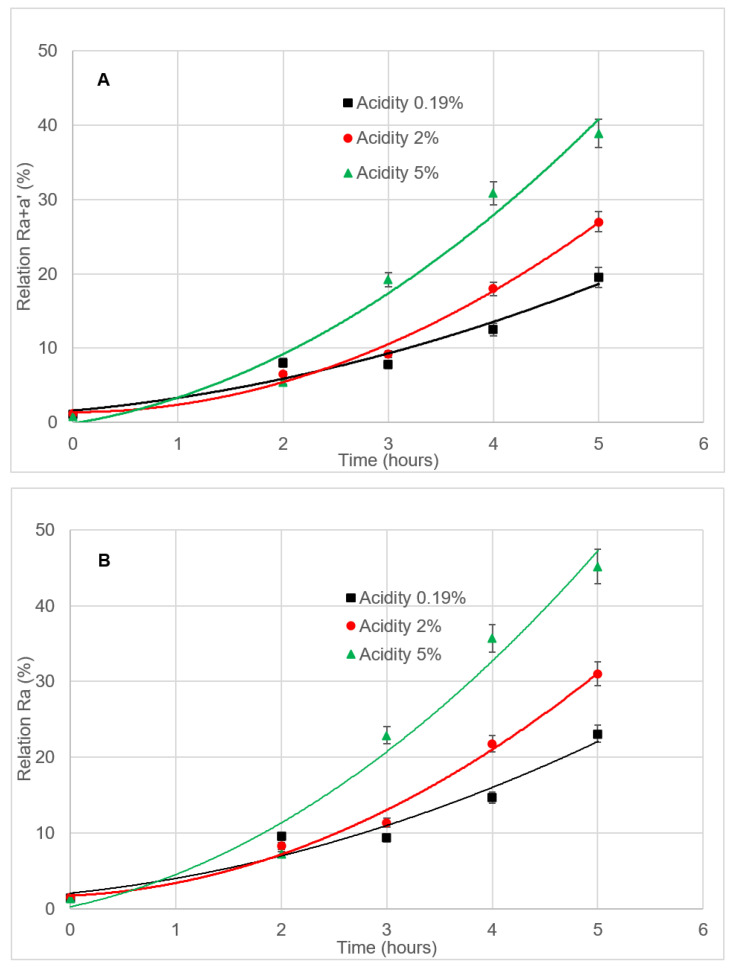
(**A**): R_a+a’_ relation vs. soft deodorization time and (**B**): R_a_ relation vs. soft deodorization length, in VOO samples from the picual cultivar of different free acidity values (5, 2 and 0.19%, from top to bottom). The soft deodorization processes were carried out at 98 °C with N_2_ as a stripping gas. Each value corresponds to the average of two individual pieces of data.

According to the data obtained, the higher the acidity, the higher the increase in the R_a_ and R_a+a’_ relations, the effect being more pronounced when *phy a’* is left aside (Figure 4), since in this case the 17% limit is reached after 1.25–2 h from the highest to the lowest acidity, instead of 1.5–2.25 h, respectively. Therefore, it is clear that high acidity enhances *phy a+a’* losses and *pyphy a* formation. Furthermore, *pyphy a* formation is clearly bound to oil quality expressed over its free fatty acid content, which contrasts with that indicated in the literature, in which a prediction model focused on olive oil shelf life stated that even if *pyphy a* is strongly related with light exposure and storage temperature, it does not show any association with oil quality nor with its chemical composition [42].

#### 3.4.4. Effect of the Stripping Gas

The study of the influence of the carrier gas on the chlorophyll pigments was carried out on hojiblanca VOO. Those samples were subjected to 2 to 5 h length deodorizations at 98 °C, using either N_2_ or air as a stripping gas (Table 1, Treatment #3).

**Figure 5:**
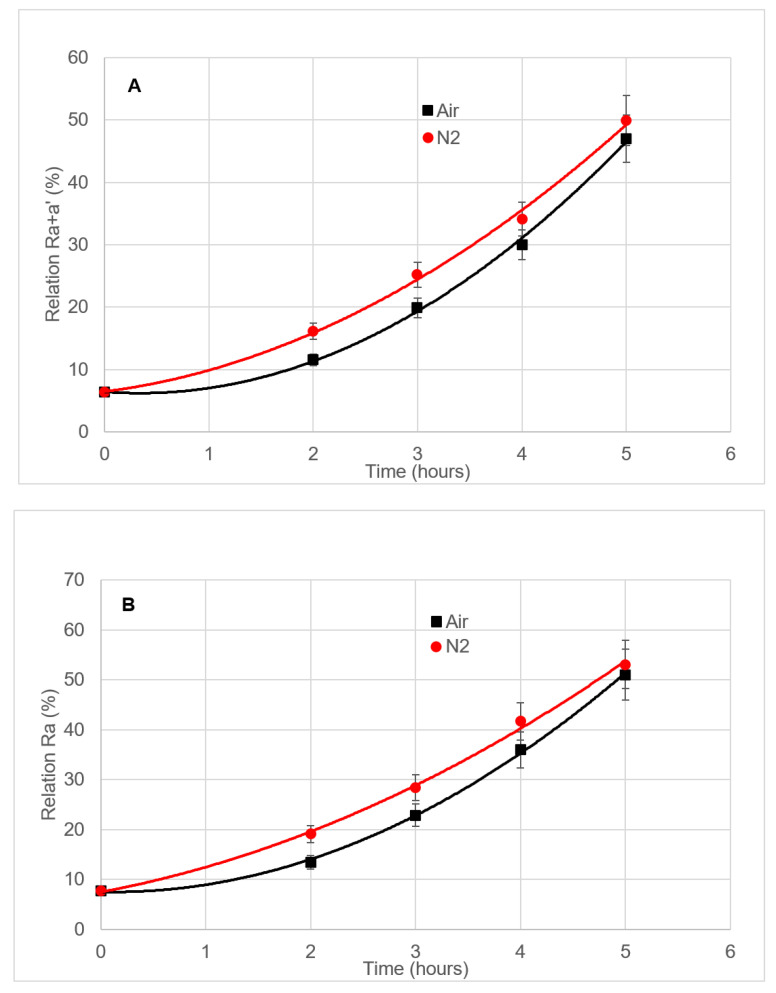
(**A**): R_a+a’_ relation vs. deodorization length and (**B**): R_a_ relation vs. deodorization length, in VOO samples from the hojiblanca cultivar. The deodorization process was carried out at 98 °C. Either N_2_ or air was used as a carrier gas. Each value corresponds to the average of two individual pieces of data.

The results are shown in Figure 5. When N_2_ is utilized as a stripping gas, the R_a+a’_ relation is around 4.6–5% higher than when air is chosen (Figure 5A), meaning that the 17% limit is exceeded after 2.0 h in the case of N_2_, and after 2.7 h if air is applied. After three hours, there is not a statistically significant difference on the R_a+a’_ relation between both stripping gases. The same tendency is observed for the R_a_ relation, although the time to surpass the limit is 1.5 and 2.4 h, respectively (Figure 5B).

#### 3.4.5. Effect of Neutralization Plus Soft Deodorization

Table 4 shows the results of applying neutralization, and neutralization followed by soft deodorization (3 h, 98 °C, N_2_ stripping gas), together with the initial pigment contents in the samples under study. VOO sample L-2 possesses the lowest amount; therefore, *pyphy a* is not formed in a substantial way. Consequently, R_a+a’_ and R_a_ equal zero. After neutralizing VOO sample M-2, *phy a+a’* and *pyphy a* content decrease minimally, which turns into an increase in R_a+a’_ and R_a_, although without substantial meaning. This is in the way round for sample H-2 but, as it may be expected, the subsequent deodorization resulted in *phy a+a’* decrease and *pyphy a* increase, with the corresponding change in the R_a+a’_ and R_a_ relations.

In no case the R_a+a’_ and R_a_ relations exceed the 17% value established as a limit from which an oil may be suspected to be soft deodorized.

## 4. Conclusions

In this pilot study, we observed that changes in chlorophyll pigments, due to soft the deodorization process, depend on the temperature, the limit of which was 100 °C. Below such ceiling, the rate at which *pyphy a* is formed is lower than the rate at which *phy a+a’* disappear. This indicates that, besides *pyphy a* formation, there exist parallel processes through which other non-detected compounds are formed. As a consequence, the R_a+a’_ and R_a_ relations are considered to be more directly linked to *phy a+a’* decrease than to *pyphy a* formation.

Stripping gas slightly affects the transformation of chlorophyll pigments; in fact, N_2_ enhances the increment in the R_a+a’_ and R_a_ relations.

Acidity also boosts the increment in the R_a+a’_ and R_a_ relations.

Regarding the oil nature, the higher the initial *phy a+a’* content, the higher the increase in the R_a+a’_ and R_a_ relations. If the initial *phy a+a’* presence is too low, the value of the R_a+a’_ and R_a_ relations will be zero.

Finally, we are sensitive to the fact that the number of samples under study was too limited to draw definite conclusions, yet it is our intention through this approach to offer a new insight in the detection of the soft deodorization oils in virgin olive oils. Indeed we will continue developing this line of research to answer open questions such as the fate of *phy (a+a’)* or the actual influence of the cultivar on the chlorophyll profiles.

## Figures and Tables

**Table 1:** Oil sample varieties and soft deodorization conditions. Each treatment was done in duplicate.

Olive Oil Variety	Treatment	Deodorization Conditions		
		Time (h)	Temperature (°C)	Gas
Hojiblanca	Treatment #1	0.5, 1.5, 2.5, 3.5, 4.5, 5.5	98	N_2_
	Treatment #2	2	50, 75, 100, 130, 150	N_2_
	Treatment #3	2, 3, 4, 5	98	Air/N_2_
Manzanilla 1	Treatment #1	0.5, 2.5, 3.5, 4.5, 5.5	98	N_2_
	Treatment #2	2	50, 75, 100, 130, 150	N_2_
Manzanilla 2	Treatment #1	0.5, 2.5, 3.5, 4.5, 5.5	98	N_2_
	Treatment #2	2	50, 75, 100, 130, 150	N_2_
Picual	Treatment #1	0.5, 2.5, 3.5, 4.5, 5.5	98	N_2_
	0.19% free acidity	2, 3, 4, 5	98	N_2_
	2% free acidity	2, 3, 4, 5	98	N_2_
	5% free acidity	2, 3, 4, 5	98	N_2_

**Table 2:** Statistical parameters for pheophytin (*a+a’*), pyropheophytin *a*, and Ra+a’ determinations in three different virgin olive oil (VOO) samples: L-1, M-1 and H-1 with low, medium and high chlorophyll concentration, respectively.

Parameters	VOO Samples		
	L-1	M-1	H-1
**Pheophytin (*a+a’*)**			
Mean (mg/kg)	**11.6**	**20.9**	**41.5**
S_r_	0.77	0.18	0.14
r	2.15	0.52	0.40
RSD_r_, % (n = 3)	6.59	0.89	0.34
CI (0.05)	0.87	0.21	0.16
**Pyropheophytin *a***			
Mean (mg/kg)	**0.1**	**0.9**	**2.0**
S_r_	0.01	0.04	0.05
r	0.03	0.11	0.15
RSD_r_, % (n = 3)	10	4.44	2.50
CI (0.05)	0.01	0.05	0.06
**R_a+a’_**			
Mean (%)	**1.1**	**3.9**	**4.6**
S_r_	0.02	0.18	0.12
r	0.04	0.50	0.32
RSD_r_, % (n = 3)	1.82	4.62	2.61
CI (0.05)	0.02	0.20	0.13

n: number of replicates; S_r_: Standard deviation of the repeatability = $\sqrt{\frac{\sum {\left(x-\bar{x}\right)}^{2}}{\left(n-1\right)}}$; RSD_r_, %: Relative standard deviation of the repeatability = 100 × S_r_/mean; r: repeatability = 2.8√S_r_^2^; CI: Confidence Interval (95%). Each value corresponds to the average of three individual data.

**Table 3:** Chlorophyll pigments (mg/kg) originally present in the olive oil samples under study together with R_a+a’_ and R_a_ (%).

Olive Oil Variety	*Phy a*	*Phy a’*	*Pyphy a*	R_a+a_	R_a_
Hojiblanca	8.74	2.16	0.70	6.12	7.52
Manzanilla 1	102.48	13.05	3.44	2.90	3.26
Manzanilla 2	14.36	1.38	0.00	0.03	0.04
Picual	10.71	10.22	2.08	0.92	0.97

*phy a*: pheophytin *a*, *phy a’*: pheophytin *a’* and *pyphy* a: pyropheophytin a. Each value corresponds to the average of two individual pieces of data.

**Table 4:** Chlorophyll derivative concentrations (mg/kg) present in olive oil samples with low (L-2), medium (M-2) and high (H-2) pigment contents after different treatments: neutralization, filtration, and soft deodorization under N_2_, at 98 °C, for 3 h. R_a+a’_ and R_a_ (both in %) are also given.

Sample	Treatment	*phy a*	*phy a’*	*pyphy a*	R_a+a’_	R_a_
L-2	Initial	2.53	0.07	0.00	0.00	0.00
	Neutralization	2.49	0.50	0.00	0.00	0.00
	Neutralization + soft deodorization	1.38	0.40	0.00	0.00	0.00
M-2	Initial	16.52	1.66	0.58	3.09	3.39 ^a^
	Neutralization	12.65	2.36	0.46	2.97	3.51 ^a^
	Neutralization + soft deodorization	11.75	2.67	0.78	5.13	6.22 ^b^
H-2	Initial	36.30	4.07	1.22	2.93	3.25 ^a^
	Neutralization	34.92	7.72	1.41	3.20	3.88 ^a^
	Neutralization + soft deodorization	17.94	3.85	2.23	9.28	11.06 ^c^

*phy a*: pheophytin *a*; *phy a’*: pheophytin *a’* and *pyphy a*: pyropheophytin *a*. Each value corresponds to the average of two individual pieces of data. ^a, b, c^Equal letters indicate that differences are not statistically significant.
